# Supplementary material for: Molecular characterization of the stress network in individuals at risk for schizophrenia
Source: Neurobiol Stress. 2021 Feb 10;14:100307. doi: 10.1016/j.ynstr.2021.100307 (PMC7893486; doi:10.1016/j.ynstr.2021.100307)
Supplement: Multimedia component 1 [file mmc1.pdf]

Supplementary Information

Molecular characterization of the stress network in individuals at risk for schizophrenia

MandyMeijer[a,b,c], ArlinKeo[a,d], Judith M.C.van Leeuwen[e,f], OlehDzyubachyk[g], Onno C.Meijer[b], Christiaan H.Vinkers[h,i,1], AhmedMahfouz[a,d,j,1,*]

[a]*Leiden Computational Biology Center, Leiden University Medical Center, 2333 ZA, Leiden, the Netherlands*
[b]*Department of Internal Medicine, Division of Endocrinology, Leiden University Medical Center, 2333 ZA, Leiden, the Netherlands*
[c]*Department of Human Genetics, Cognition and Behaviour, Donders Institute for Brain, Radboud University Medical Center, 6525 GA, Nijmegen, the Netherlands*
[d]*Delft Bioinformatics Lab, Delft University of Technology, 2628 XE, Delft, the Netherlands*
[e]*University Medical Center Utrecht, Department of Psychiatry, Utrecht, the Netherlands*
[f]*Donders Institute for Brain, Cognition, and Behaviour, Radboud University Medical Center, Nijmegen, the Netherlands*
[g]*Department of Radiology, Division of Medical Image Processing, Leiden University Medical Center, 2333 ZA, Leiden, the Netherlands*
[h]*Department of Psychiatry, Amsterdam UMC (location VUmc)/GGZ InGeest, 1081 HV, Amsterdam, the Netherlands*
[i]*Department of Anatomy and Neurosciences, Amsterdam UMC (location VUmc), 1081 HV, Amsterdam, the Netherlands*
[j]*Department of Human Genetics, Leiden University Medical Center, 2333ZA, Leiden, the Netherlands*

[*]Corresponding author. Einthovenweg 20, 2333ZC, Leiden, the Netherlands.
E-mail address: a.mahfouz@lumc.nl (A. Mahfouz)

[1]Shared last authorship.

Differentially expressed genes in our stress network based on siblings of schizophrenia patients

We identified the gene expression signatures of our stress network with altered stress-induced activity in siblings of schizophrenia patients (from now on referred to as 'our stress network') by determining which genes are differentially expressed in our stress network compared to the rest of the brain minus the cerebellum. Using a meta-analysis approach to combine results across all donors of the AHBA (n=6), we identified 261 differentially expressed genes (BH-adjusted $p < 0.05$ and $\log_2$(fold-change) > |1|, **Figure S1; Table S2**). Among those genes, 229 were higher expressed, while the other 32 genes were lower expressed in the stress network compared to the rest of the brain minus the cerebellum. Using a bootstrapping approach (see Methods, Main Text), we found the identified set of genes to be highly robust to the imbalance between the number of AHBA samples inside and outside our stress network (61% of our initial 261 genes were differentially expressed in at least 95% of all 1000 iterations).

As a sensitivity analysis, we next selected genes that were differentially expressed (BH-adjusted $p < 0.05$ and $\log_2$(fold-change) > |1|) in at least five out of the six donors. This more stringent approach resulted in a smaller set of 63 differentially expressed genes (**Figure S2**). The smaller set of 63 genes was entirely contained within the larger set of 261 genes obtained by the meta-analysis, therefore we choose to perform subsequent analyses on the larger gene set.

The differentially expressed genes in the brain regions vulnerable to stress in the siblings of schizophrenia patients showed an opposite expression pattern in the cerebellum compared to the rest of the brain (**Figure S1B**), supporting our approach of excluding the cerebellum from our analysis (see Methods).

To assess whether our results are driven by the higher representation of cerebral cortex samples in the brain regions vulnerable to stress in siblings of schizophrenia patients (109 out of 127; 91%) compared to the rest of the brain (1,950 out of 3,225; 60%), we identified differentially expressed genes within each anatomically-defined region (hippocampal formation, cerebellum, frontal gyrus,

temporal gyrus and cingulate gyrus) separately (**Table S3**). Due to the small sample size, it was not possible to assess differential gene expression between brain regions vulnerable to stress based on healthy siblings of schizophrenia patients compared to the rest of the brain minus the cerebellum within each anatomically-defined region (**Table S4**).

The differentially expressed genes consistently showed high expression values in the cortex but not the hippocampus, and mostly low expression levels in non-cortical areas (**Figure S1D and Figure S3**). The two most differentially expressed genes in the stress-specific brain regions are D-amino acid oxidase (*DAO)* (BH-adjusted p-value = $4.99*10^{-17}$, $log_2(FC) = -2.40$) and Transmembrane protein 155 (*TMEM155)* (BH-adjusted p-value = $4.90*10^{-55}$, $log_2(FC) = 3.11$). *TMEM155* is highly expressed in the brain, specifically in the cerebral cortex and claustrum (**Figure S4**).[35] Its biological function, however, remains unknown, largely because there are no orthologs in the Mus musculus or the Rattus norvegicus. *TMEM155* orthologs are, however, found in chimpanzee[36] and rhesus macaque[37], with similar brain expression patterns between the human and macaque (**Figure S4B and S4C**).

Functionality and cell type-specificity of differentially expressed genes in the stress network based on siblings of schizophrenia patients

A GO term enrichment analysis was performed to assess whether the differentially expressed genes in our stress network are enriched for specific functions. The differentially expressed genes were enriched for GO terms involved in neuronal development and neurogenesis, synaptic signal transmission, learning and memory, behavior and glutamate receptor signaling (**Figure S5A and Table S2**). Genes involved in most processes based on GO terms (at least assigned to five out of twelve GO terms) include *CRH, MEF2C, NETO1, PLK2, NEUROD2, NR2E1, SERPINF1, CHRNA3, ISLR2, CUX1* and *TBR1*. Enrichment analysis for cellular components indicated that the proteins coded by the differentially expressed genes were mainly found at the synapse, reflecting the high expression of the genes in the synapse-dense cerebral cortex. To control for the case that the results of the GO term

enrichment analysis are solely driven by genes already known to be associated with stress-related disorders, the analysis was also performed after exclusion of 58 genes associated to stress related disorders. We found still very similar results, indicating that differentially expressed genes in the brain regions vulnerable to stress based on siblings of schizophrenia patients are predominantly involved in genes relevant for stress-related diseases but not in non-brain-related disorders and traits.

Next, we identified the specific cell types underlying the differential gene expression levels in our stress network using enrichment analysis of cortical cell type markers.[23] Enrichment was found for neuronal cell markers (p = 0.0414, BH corrected), including *WIF1, CHRNA3, TAC3, VIP, C1QL1, SLITRK6, NEUROD6, KCTD16, TBR1, CREG2* and *DLX1*. The list of differentially expressed genes included a few astrocytes markers (*AQP9, EMX2, KIRREL2, NR2E1* and *LHX2*) and oligodendrocytes (*EBF3*), but was not significantly enriched (p = 0.4202; p = 0.5267, BH corrected). Moreover, we found that neuronal markers showed a partially overlapping distribution in a t-Distributed Stochastic Neighbor Embedding (t-SNE) map of all genes across the whole brain as the differentially expressed genes in, indicating that neuronal markers and the differentially expressed genes show the same expression patterns across cortical areas (**Figure S5B**) and thus differential activity may depend on neuronal gene expression.

We further assessed whether the set of differentially expressed genes was particularly expressed in a neuronal subtype. Interestingly, five out of the 22 known thalamic cholinergic neuron markers were found in the set of the differentially expressed genes (*CHRNA3* (BH-adjusted p-value = $6.6*10^{-6}$, $\log_2$(FC) = -1.47), *IRX2* (BH-adjusted p-value = $1.31*10^{-}42$, log2(FC) = -2.03), *NPPA* (BH-adjusted p-value = $1.85*10^{-34}$, log2(FC) = 1.89), *TYRP1* (BH-adjusted p-value = $3.3*10^{-14}$, $\log_2$(FC) = 1.1) and *ZIC4* (BH-adjusted p-value = $7..57*10^{-8}$; $\log_2$(FC) = -1.24), BH-adjusted p-value = $3.3*10^{-4}$; **Figure S5C**). This finding in thalamic cholinergic neuron markers is independent of the number of thalamic samples inside and outside the brain regions vulnerable to stress, showing that this result is not biased by the number of thalamic samples involved in the maladaptive stress response of healthy siblings of schizophrenia patients. Additionally, some of the differentially expressed genes are reported to be part of the

molecular fingerprint of particular cell types in the human cortex.[38] For example, *RORB* represents a subclass of excitatory neurons present in the cortex and was modestly enriched in differentially activated brain regions. *VIP* represents one of the four main subclasses of inhibitory cortical neurons and was quite substantially overrepresented (BH-adjusted p-value = $5.73*10^{-69}$, $\log_2$(FC) = 1.66). Markers for smaller subsets of inhibitory neurons included overrepresentation of *NPY* (a small set of deep projecting inhibitory layer 3 neurons; BH-adjusted p-value = $3.53*10^{-25}$, $\log_2$(FC) = 1.60) and *SERPINF1* (a subset of inhibitory neurons in layer 1; BH-adjusted p-value = $7.83*10^{-37}$, $\log_2$(FC) = 1.27), and underrepresentation of *CBLN1* (a subset of VIP expressing inhibitory neurons in layer 4; BH-adjusted p-value = $1.85*10^{-11}$, $\log_2$(FC) = 1.20). Using a human-specific single cell RNA-sequencing data of the medio-temporal gyrus[25], we found the differentially expressed genes to be mainly enriched in glutamatergic excitatory neurons compared to GABAergic and non-neuronal cells, using a Wilcoxon rank test (p-value = $2.2*10^{-}16$, **Figure S5D**).

Differentially expressed genes in our stress network based on siblings of schizophrenia patients are associated to stress-related diseases

We hypothesized that the differentially expressed genes in our stress network would be associated to the genetic background of psychiatric disorders not limited to schizophrenia, particularly for stress-related brain disorders, as stress plays a major role in the development of these disorders. Using genetic variants from GWAS of the Genomics of Psychiatry Consortium[26, 27], we assessed whether schizophrenia-associated risk loci are enriched in the set of differentially expressed genes. Indeed, schizophrenia risk genes were enriched in the differentially expressed genes in our stress network (Fisher Exact test, BH-adjusted p-value = $2.4*10^{-3}$). The schizophrenia risk genes *KCNV1, DOC2A, NGEF, NRGN, MEF2C, BCL11b, SATB2, FAM5B, CHRM3, CHRM4* and *TAC3* were present in our differentially expressed genes, and all except one (*CRHM4*) were higher expressed in the brain regions vulnerable to stress in siblings of schizophrenia patients. Based on a recent GWAS across multiple psychiatric disorder, multiple pleiotropic risk genes were identified.[39] Differentially expressed genes in our stress

network were significantly enriched in the set of pleiotropic risk genes for psychiatric disorders (Fisher Exact test, BH-adjusted p-value = 0.0065, OR = 30.3), including *KCNQ5, BCL11b, NGEF, CHRNA3, NRGN,* and *CHRM4*. Furthermore, gene-disease associations from DisGeNet, a manually curated database, were used to assess risk gene enrichment for psychiatric, brain and non-brain diseases and non-disease traits. Enrichment was found for neuropsychiatric disorders (schizophrenia, schizoaffective disorder, major depressive disorder, bipolar disorder, and anxiety disorders) and other brain diseases (epilepsy). However, no gene enrichment was found for non-brain diseases (e.g. osteoporosis) and non-disease traits (e.g. height and waste-hip-ratio; **Figure S6**). Thus, differentially expressed genes in our stress network are predominantly involved in genes relevant for stress-related diseases not limited to schizophrenia, but not in non-brain-related disorders and traits.

Interestingly, the set of 261 differentially expressed genes in our stress network included a considerable number of neuropeptides and receptors. Apart from their use as markers for specific cell types (e.g. NPY, VIP, and CCK[38]), these are important for signaling in the brain (e.g. NPY stimulates the release of CRH in the hypothalamus[40]) and some of them are known to be involved in the regulation of stress.[41, 42] Therefore, we assessed whether there were more neuropeptides and receptors in our set of genes than you would expect by chance. For both the neuropeptides and the receptors, we found higher odds ratios for brain and psychiatric disorders, with the biggest effect sizes in psychiatric disorders (**Figure S6**). Effect sizes for receptor enrichment in major depressive disorder and bipolar disorder were significant (p-value = 0.0003, OR = 1.86 and p-value < 0.00001, OR = 2.57, respectively).

Cortisol sensitivity of our stress network based on siblings of schizophrenia patients

The enrichment of the neuronal GO terms in our set of genes and the association with stress-related diseases indicates that the differentially expressed genes in our stress network are relevant for stress in general and may be responsive to the pivotal stress hormone cortisol. To investigate glucocorticoid sensitivity, we compared our list of differentially expressed genes with genes that show a DNA binding

site for the glucocorticoid- and/or mineralocorticoid receptors (GR and MR) in the rat hippocampus by Chromatin Immunoprecipitation sequencing after stimulation with the endogenous steroid corticosterone.[34] Differentially expressed genes that showed DNA binding loci for the GR exclusively are: *STON1, HS3ST2, HTR2A, ZNF831, CACNG3, NPTX2, EPHB6, LRRC7, KCNC2, HTR1A, NETO1, CYP26A1, NCALD, EMX2, CXCL14* and *RORB* (16/704 genes with binding sites, BH-adjusted p- value = $2.97*10^{-2}$). The differentially expressed genes *HSPB3, EGR3, NEUROD2, TBR1, SLC26A6, SLIT1, PCDH20, MAST3, BCL11b, SCN3B, TFAP2B, MAB21L1* and *CHN1* have DNA binding loci for both the GR and the MR (13/459 genes with binding sites, BH-adjusted p-value = $9.8*10^{-3}$). There was no significant MR DNA binding loci enrichment (*IRX2, IRX3, PIRT, CBLN1, KCNV1, KCNS1, ICAM5, FHL2, DLGAP, ST6GALNAC5, RFTN1, KCNQ5, KCNH1* and *LMO7*; 14/1247 genes with binding sites, BH-adjusted p-value = 0.46). These results indicate that differentially expressed genes in our stress network are enriched for DNA-binding loci of GR but not MR, further consolidating the relevance of these brain regions in aftermath of the responses to acute stress when the GR plays a dominant role.

Putative molecular pathway as the basis for inter-individual differences in stress reactivity

To create a more comprehensive possible pathway for the molecular mechanisms underlying human stress reactivity, we performed a thorough literature search on PubMed for all differentially expressed genes to assess whether previous studies have found these genes to be regulated by or regulating the HPA-axis. We found that 53 of our differentially expressed genes were earlier described to interact with the HPA-axis (**Table S5**), with a subset of 36 genes reported to interact with each other (F**igure S7, Table S5**). A putative pathway with factors involved in stress reactivity in siblings of schizophrenia patients was build based on these 36 genes (**Figure S7**).

Table S3 Differentially expressed genes in the cerebellum and hippocampus

	Gene	BH-adjusted p-value	$Log_2(FC)$
Cerebellum (5 samples inside and 78 samples outside the stress-vulnerability related brain regions)	*SLN*	$1.7*10^{-3}$	1.13
	CCDC155	$1.3*10^{-3}$	1.01
	SEMA3C	$4.0*10^{-3}$	1.01
	NTNG1	$1.7*10^{-3}$	-1.18
	CHRNB3	$2.1*10^{-3}$	-1.35
	SHB	$6.0*10^{-4}$	-1.40
Hippocampus (19 samples inside and 26 samples outside the stress-vulnerability related brain regions)	*ZMAT4*	$1.04*10^{-10}$	1.30
	SLC17A6	$1.6*10^{-4}$	1.11
	PACAP	$2.35*10^{-5}$	1.05
	IGSF3	$1.01*10^{-7}$	1.03
	DMKN	$5.07*10^{-9}$	-1.12
	IQGAP3	$4.3*10^{-3}$	-1.15
	SCGN	$5.57*10^{-9}$	-1.48

Table S4 Distribution of samples of the Allen Human Brain Atlas

	Donor 1	Donor 2	Donor 3	Donor 4	Donor 5	Donor 6	*Not in mask*	Note
Superior frontal gyrus	4	6	1	1	3	3	3	Cortex
Middle frontal gyrus	1	1	1	0	1	1	5	Cortex
Inferior frontal gyrus	2	3	0	1	0	0	7	Cortex
Parolfactory gyrus	0	1	0	0	0	0	1	Cortex
Superior rostral gyrus	1	1	0	0	0	0	1	Cortex
Supramarginal gyrus	1	1	0	1	2	0	4	Cortex
Angular gyrus	0	0	1	0	1	1	4	Cortex
Precuneus	1	0	1	0	0	1	5	Cortex
Superior temporal gyrus	4	2	1	1	0	2	5	Cortex
Middle temporal gyrus	7	1	1	0	0	0	4	Cortex
Heschl's gyrus	0	0	0	1	0	0	1	Cortex
Transverse gyrus	0	0	0	0	1	0	1	Cortex
Planum temporale	2	0	0	1	0	0	1	Cortex
Planum polare	1	1	2	2	1	1	1	Cortex
Cuneus	2	0	0	0	0	0	5	Cortex
Anterior cingulate gyrus	0	3	1	0	4	2	2	Cortex
Parietal cingulate gyrus	0	0	1	2	1	1	4	Cortex
Hippocampal formation	5	3	2	4	2	3	26	
Claustrum	0	1	1	0	2	0	0	
Caudate nucleus	0	1	0	0	1	0	89	
Lateral group of nuclei	0	3	0	1	1	0	0	
Cerebellum	3	2	0	0	0	0	78	Excluded

Red = <2 samples in the donors
Orange = <2 samples outside the mask
Green = comparison was possible, but effect sizes were too small

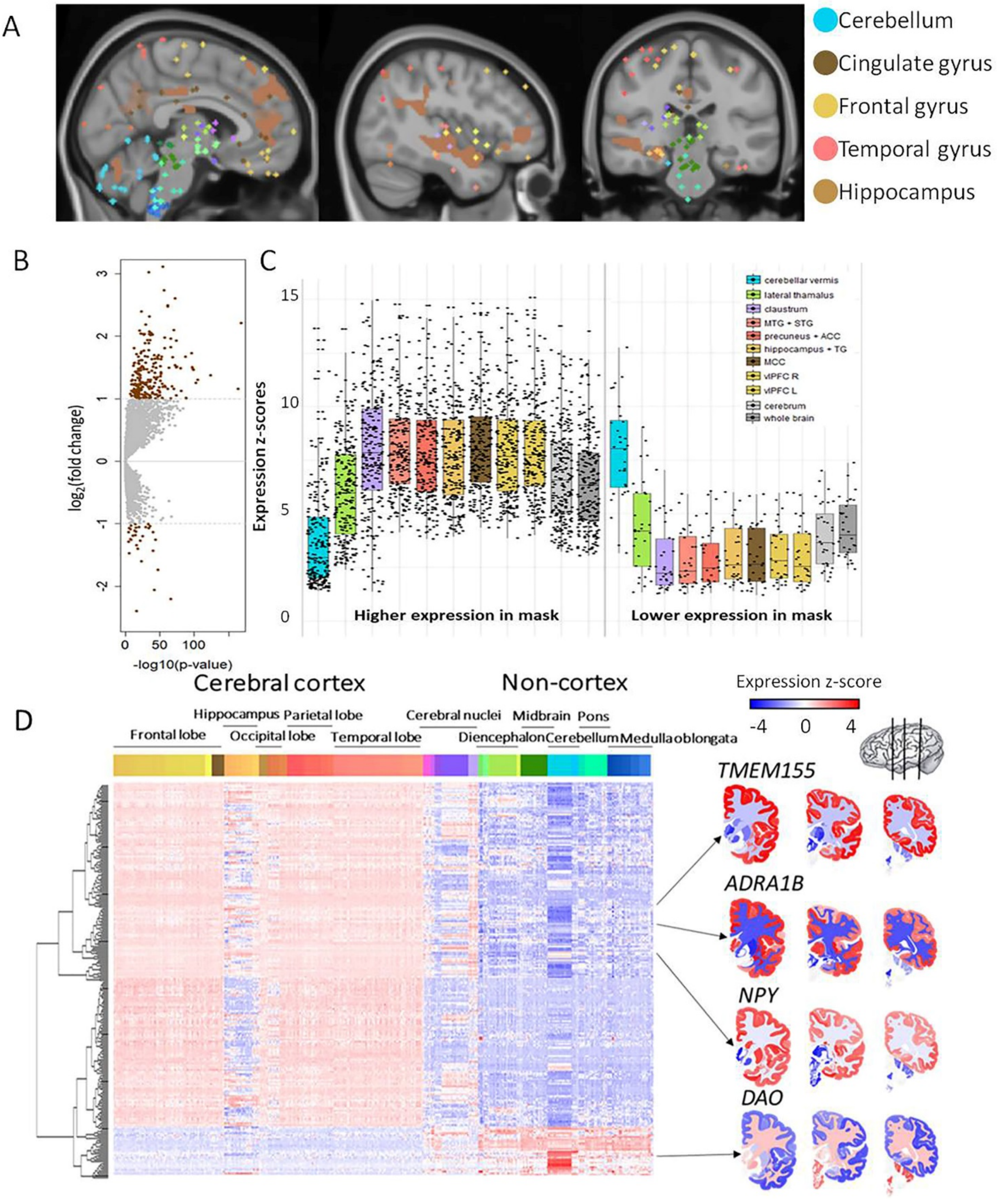

Figure S1 Differentially expressed genes in brain regions vulnerable to stress based on siblings of schizophrenia patients can be identified using gene expression atlases. (A) Brain regions in our stress network are present throughout the brain (including cerebellum, cingulate gyrus, frontal gyrus, temporal gyrus, and hippocampal formation). For the analysis, all regions were combined. (B) Differential gene expression was determined for our stress network compared to the rest of the brain minus cerebellum. Significant genes (BH-adjusted p-value < 0.05 & $\log_2$(fold-change) > |1|) have higher

expression in our stress network. Grey dots represent non-significant genes and brown dots represent significant genes based on meta-analysis across all six AHBA donors. (C) The box plots show the expression of the higher (left) and lower (right) expressed genes compared to the rest of the brain minus cerebellum in the brain regions of interest from the stress network. (D) In the whole brain, differentially expressed genes show mostly high expression levels in the cortex and low expression levels in non-cortical brain regions. In the heatmap, each row represents a gene and each column represents a sample and all samples of the AHBA are illustrated here. On the right, coronal brain sections for the genes *TMEM155*, *ADRA1B*, *NPY* and *DAO* are presented. Colors indicates high (red) and low (blue) expression levels.

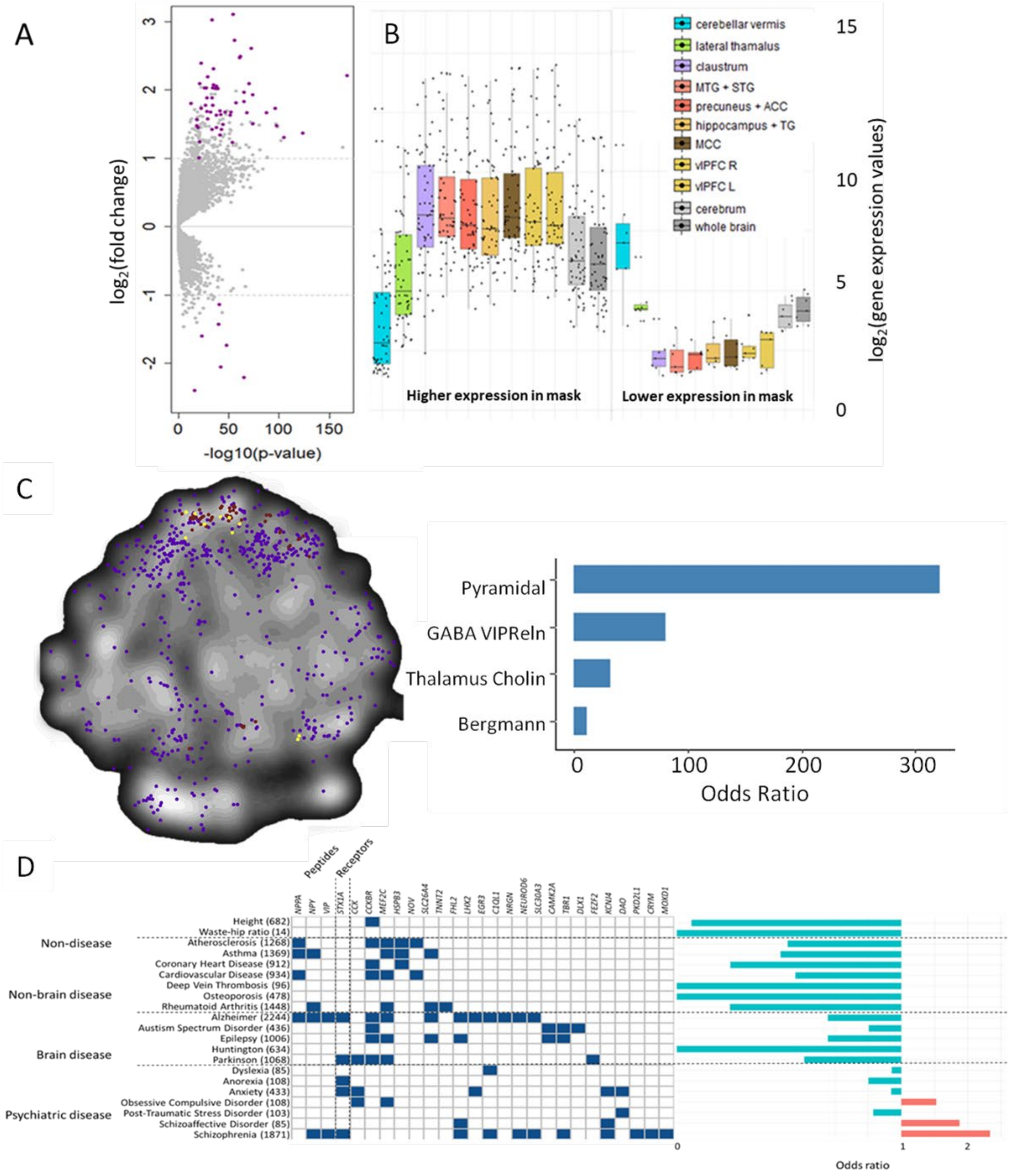
A
log2(fold change)
-log10(p-value)
B
cerebellar vermis
lateral thamalus
claustrum
MTG + STG
precuneus + ACC
hippocampus + TG
MCC
vlPFC R
vlPFC L
cerebrum
whole brain
Higher expression in mask
Lower expression in mask
log2(gene expression values)
C
Pyramidal
GABA VIPReln
Thalamus Cholin
Bergmann
Odds Ratio
D
Peptides
Receptors
NPPA
NPY
VIP
STX1A
CCK
CCKBR
MEF2C
HSPB3
NOV
SLC26A4
TNNT2
FHL2
LHX2
EGR3
C1QL1
NRGN
NEUROD6
SLC30A3
CAMK2A
TBR1
DLX1
FEZF2
KCNJ4
DAO
PKD2L1
CRYM
MOXD1
Non-disease
Height (682)
Waste-hip ratio (14)
Non-brain disease
Atherosclerosis (1268)
Asthma (1369)
Coronary Heart Disease (912)
Cardiovascular Disease (934)
Deep Vein Thrombosis (96)
Osteoporosis (478)
Rheumatoid Arthritis (1448)
Brain disease
Alzheimer (2244)
Austism Spectrum Disorder (436)
Epilepsy (1006)
Huntington (634)
Parkinson (1068)
Psychiatric disease
Dyslexia (85)
Anorexia (108)
Anxiety (433)
Obsessive Compulsive Disorder (108)
Post-Traumatic Stress Disorder (103)
Schizoaffective Disorder (85)
Schizophrenia (1871)
Odds ratio

Figure S2 Using a stricter method of selecting differentially expressed genes results in similar outcome as using a meta-analysis. (A) Differential gene expression was determined for our stress network compared to the rest of the cerebrum for each of the six donors separately. Genes showing significance (BH-adjusted p-value < 0.05 & $\log_2$(fold-change) > |1|) in at least 5 out of the 6 donors were selected and are shown in purple in the volcano plot of the meta-analysis. Grey dots represent non-significant genes. The box plots on the right show the expression of the individual differentially expressed genes relative to the average gene expression in individual brain regions. (B) Differentially expressed genes (brown), neuronal marker genes (purple) and overlapping genes (pink) are plotted in a t-Distributed Stochastic Neighbor Embedding (t-SNE) plot acquired from BrainScope.nl[21], visualized in a heatmap of genes, where close points represent genes with similar gene expression profiles. ORs for different neuronal subtypes are presented. Neuronal subtypes without a marker represented in the differentially expressed genes are not illustrated in the graph. No significant enrichment for neuronal markers was observed. (C) Disease-risk gene enrichment was performed for the differentially expressed genes. The diseases are clustered as non-brain related disease, brain disease and psychiatric disorders. As a none-disease-associated set of genes, waste-hip ratio and height were used. The numbers between the parenthesis indicate the number of genes known to be associated with the disease based on DisGeNet. The effect size of the gene enrichment is presented on the right side of the figure, though none of them was significant.

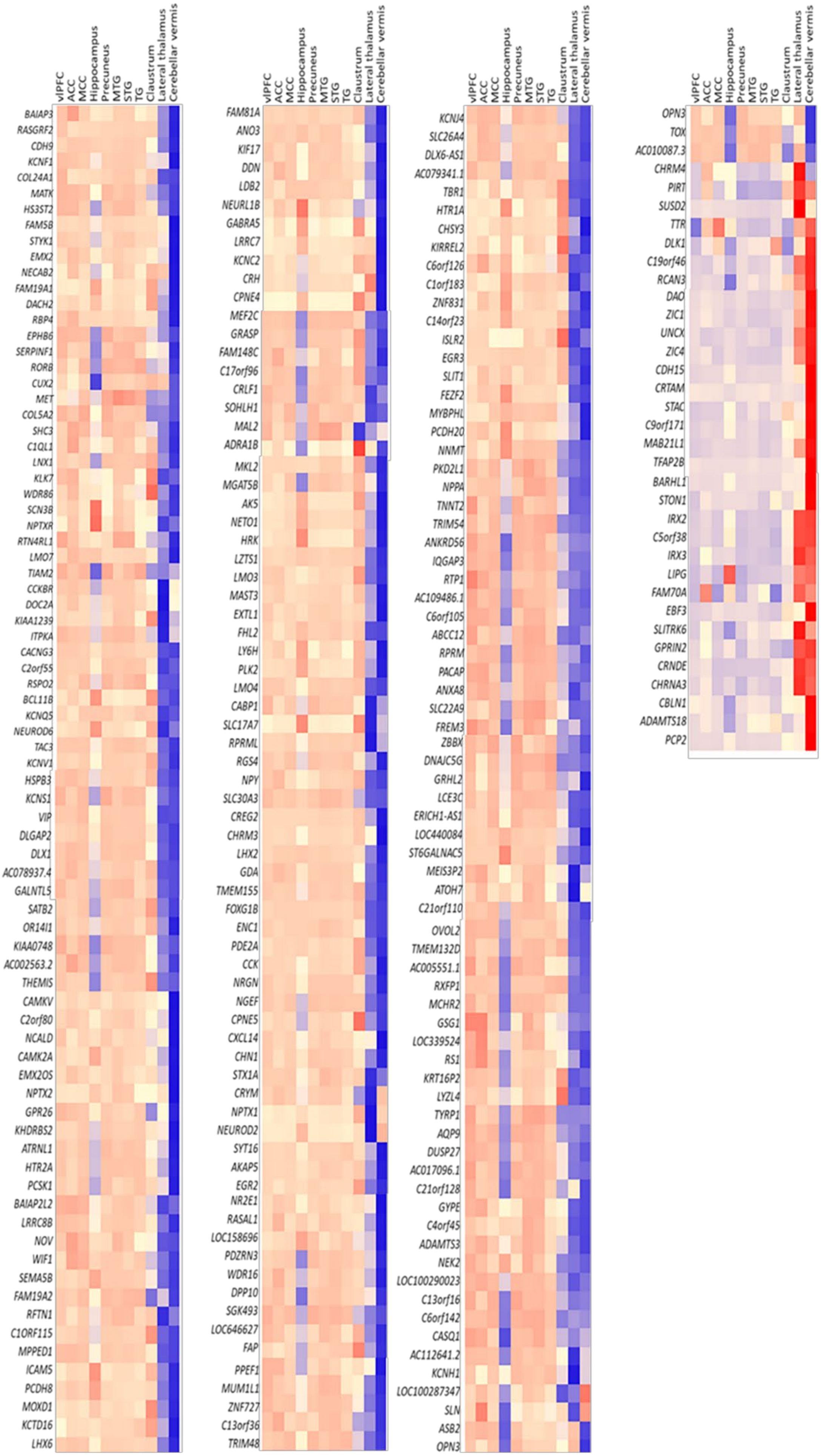

vlPFC
ACC
MCC
Hippocampus
Precuneus
MTG
STG
TG
Claustrum
Lateral thalamus
Cerebellar vermis
BAIAP3
RASGRF2
CDH9
KCNF1
COL24A1
MATK
HS3ST2
FAM5B
STYK1
EMX2
NECAB2
FAM19A1
DACH2
RBP4
EPHB6
SERPINF1
RORB
CUX2
MET
COL5A2
SHC3
C1QL1
LNX1
KLK7
WDR86
SCN3B
NPTXR
RTN4RL1
LMO7
TIAM2
CCKBR
DOC2A
KIAA1239
ITPKA
CACNG3
C2orf55
RSPO2
BCL11B
KCNQ5
NEUROD6
TAC3
KCNV1
HSPB3
KCNS1
VIP
DLGAP2
DLX1
AC078937.4
GALNTL5
SATB2
OR14I1
KIAA0748
AC002563.2
THEMIS
CAMKV
C2orf80
NCALD
CAMK2A
EMX2OS
NPTX2
GPR26
KHDRBS2
ATRNL1
HTR2A
PCSK1
BAIAP2L2
LRRC8B
NOV
WIF1
SEMA5B
FAM19A2
RFTN1
C1ORF115
MPPED1
ICAM5
PCDH8
MOXD1
KCTD16
LHX6
vlPFC
ACC
MCC
Hippocampus
Precuneus
MTG
STG
TG
Claustrum
Lateral thalamus
Cerebellar vermis
FAM81A
ANO3
KIF17
DDN
LDB2
NEURL1B
GABRA5
LRRC7
KCNC2
CRH
CPNE4
MEF2C
GRASP
FAM148C
C17orf96
CRLF1
SOHLH1
MAL2
ADRA1B
MKL2
MGAT5B
AK5
NETO1
HRK
LZTS1
LMO3
MAST3
EXTL1
FHL2
LY6H
PLK2
LMO4
CABP1
SLC17A7
RPRML
RGS4
NPY
SLC30A3
CREG2
CHRM3
LHX2
GDA
TMEM155
FOXG1B
ENC1
PDE2A
CCK
NRGN
NGEF
CPNE5
CXCL14
CHN1
STX1A
CRYM
NPTX1
NEUROD2
SYT16
AKAP5
EGR2
NR2E1
RASAL1
LOC158696
PDZRN3
WDR16
DPP10
SGK493
LOC646627
FAP
PPEF1
MUM1L1
ZNF727
C13orf36
TRIM48
vlPFC
ACC
MCC
Hippocampus
Precuneus
MTG
STG
TG
Claustrum
Lateral thalamus
Cerebellar vermis
KCNJ4
SLC26A4
DLX6-AS1
AC079341.1
TBR1
HTR1A
CHSY3
KIRREL2
C6orf126
C1orf183
ZNF831
C14orf23
ISLR2
EGR3
SLIT1
FEZF2
MYBPHL
PCDH20
NNMT
PKD2L1
NPPA
TNNT2
TRIM54
ANKRD56
IQGAP3
RTP1
AC109486.1
C6orf105
ABCC12
RPRM
PACAP
ANXA8
SLC22A9
FREM3
ZBBX
DNAJC5G
GRHL2
LCE3C
ERICH1-AS1
LOC440084
ST6GALNAC5
MEIS3P2
ATOH7
C21orf110
OVOL2
TMEM132D
AC005551.1
RXFP1
MCHR2
GSG1
LOC339524
RS1
KRT16P2
LYZL4
TYRP1
AQP9
DUSP27
AC017096.1
C21orf128
GYPE
C4orf45
ADAMTS3
NEK2
LOC100290023
C13orf16
C6orf142
CASQ1
AC112641.2
KCNH1
LOC100287347
SLN
ASB2
OPN3
vlPFC
ACC
MCC
Hippocampus
Precuneus
MTG
STG
TG
Claustrum
Lateral thalamus
Cerebellar vermis
OPN3
TOX
AC010087.3
CHRM4
PIRT
SUSD2
TTR
DLK1
C19orf46
RCAN3
DAO
ZIC1
UNCX
ZIC4
CDH15
CRTAM
STAC
C9orf171
MAB21L1
TFAP2B
BARHL1
STON1
IRX2
C5orf38
IRX3
LIPG
FAM70A
EBF3
SLITRK6
GPRIN2
CRNDE
CHRNA3
CBLN1
ADAMTS18
PCP2

Figure S3 Differential gene expression in the whole brain stress network based on siblings of schizophrenia patients. For each brain region of our defined stress network, relative gene expression levels are shown. Colors indicates high (red) or low (blue) expression.

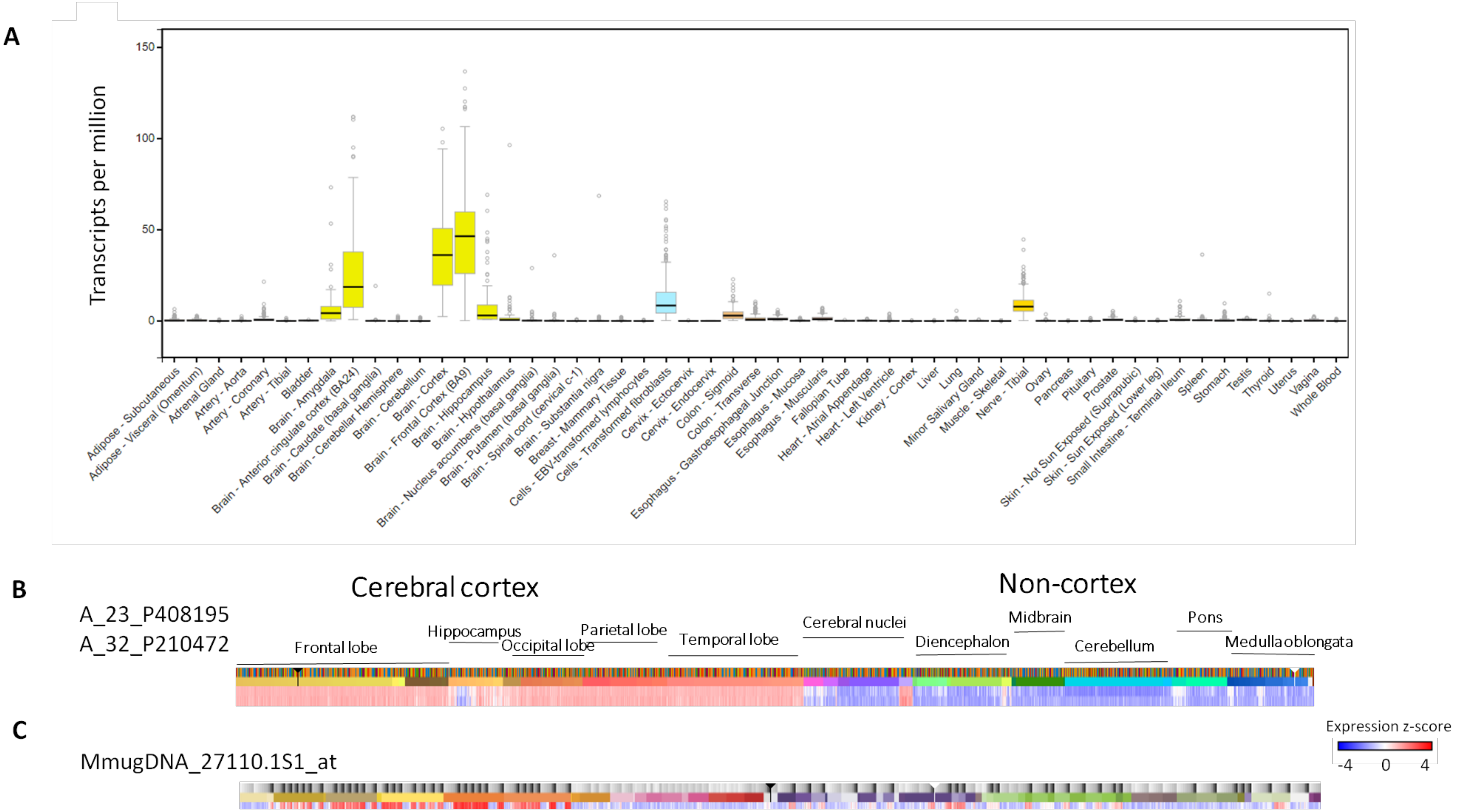
A
Transcripts per million
150
100
50
0
Adipose - Subcutaneous
Adipose - Visceral (Omentum)
Adrenal Gland
Artery - Aorta
Artery - Coronary
Artery - Tibial
Bladder
Brain - Amygdala
Brain - Anterior cingulate cortex (BA24)
Brain - Caudate (basal ganglia)
Brain - Cerebellar Hemisphere
Brain - Cerebellum
Brain - Cortex
Brain - Frontal Cortex (BA9)
Brain - Hippocampus
Brain - Hypothalamus
Brain - Nucleus accumbens (basal ganglia)
Brain - Putamen (basal ganglia)
Brain - Spinal cord (cervical c-1)
Brain - Substantia nigra
Breast - Mammary Tissue
Cells - EBV-transformed lymphocytes
Cells - Transformed fibroblasts
Cervix - Ectocervix
Cervix - Endocervix
Colon - Sigmoid
Colon - Transverse
Esophagus - Gastroesophageal Junction
Esophagus - Mucosa
Esophagus - Muscularis
Fallopian Tube
Heart - Atrial Appendage
Heart - Left Ventricle
Kidney - Cortex
Liver
Lung
Minor Salivary Gland
Muscle - Skeletal
Nerve - Tibial
Ovary
Pancreas
Pituitary
Prostate
Skin - Not Sun Exposed (Suprapubic)
Skin - Sun Exposed (Lower leg)
Small Intestine - Terminal Ileum
Spleen
Stomach
Testis
Thyroid
Uterus
Vagina
Whole Blood
B
Cerebral cortex
Non-cortex
A_23_P408195
A_32_P210472
Frontal lobe
Hippocampus
Occipital lobe
Parietal lobe
Temporal lobe
Cerebral nuclei
Diencephalon
Midbrain
Cerebellum
Pons
Medulla oblongata
C
MmugDNA_27110.1S1_at
Expression z-score
-4
0
4

Figure S4 *TMEM155* is highly expressed in the claustrum. (A) In the human body, *TMEM155* has only been identified in the brain. (B) In the human brain, *TMEM155* (measured using two probes: A_23_P408195 and A_32_P210472) is highly expressed in the cortex and claustrum. (C) The expression pattern of *TMEM155* (measured using one probe: MmugDNA_27110.1S1_at) in the macaque brain resembles the human expression pattern.

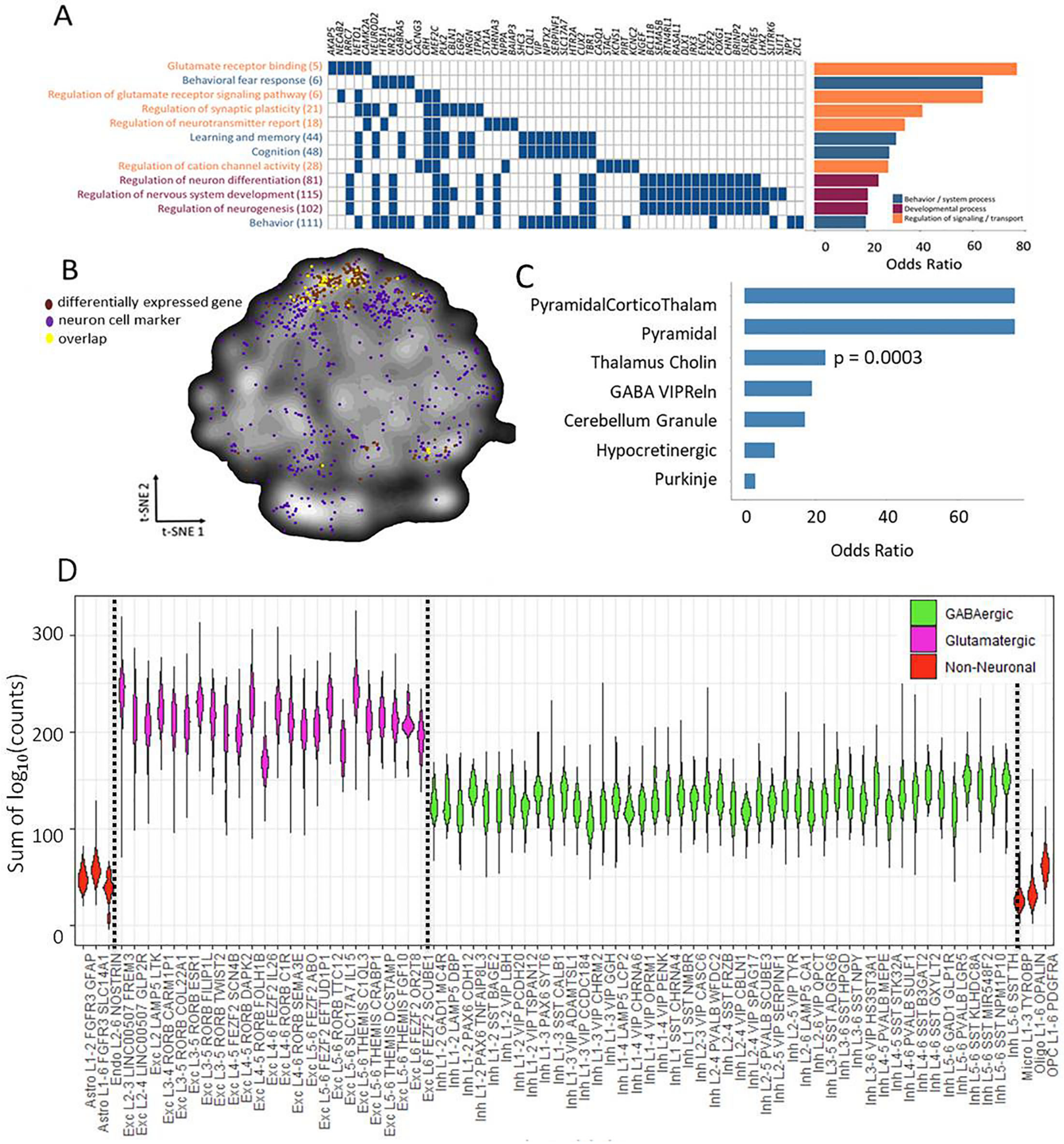

Figure S5 Functionality of differentially expressed genes in our stress network based siblings of schizophrenia patients. (A) Differentially expressed genes annotated to one of the GO terms were assigned to multiple GO terms and thus involved in multiple processes. Between parenthesis, the total number of genes assigned to the GO term is depicted. On the right side of the graph, ORs are displayed

per GO term. (B) Differentially expressed genes (brown), neuronal marker genes (purple) and overlapping genes (yellow) are plotted in a t-SNE plot generated using BrainScope.nl[21], where points close together represent genes with similar gene expression profiles. The differentially expressed genes show a similar profile in the t-SNE plot as neuronal cell markers (purple). (C) ORs for different neuronal subtypes. ORs were considered significant when the BH-adjusted p-value < 0.05 (*). Neuronal subtypes without a marker represented in the differentially expressed genes are not illustrated in the graph. (D) The sum of the log_{10} values of the counts per gene is plotted for each cell cluster. Green clusters belong to GABAergic cells, purple clusters to glutamatergic cells and red clusters to non-neuronal cells.

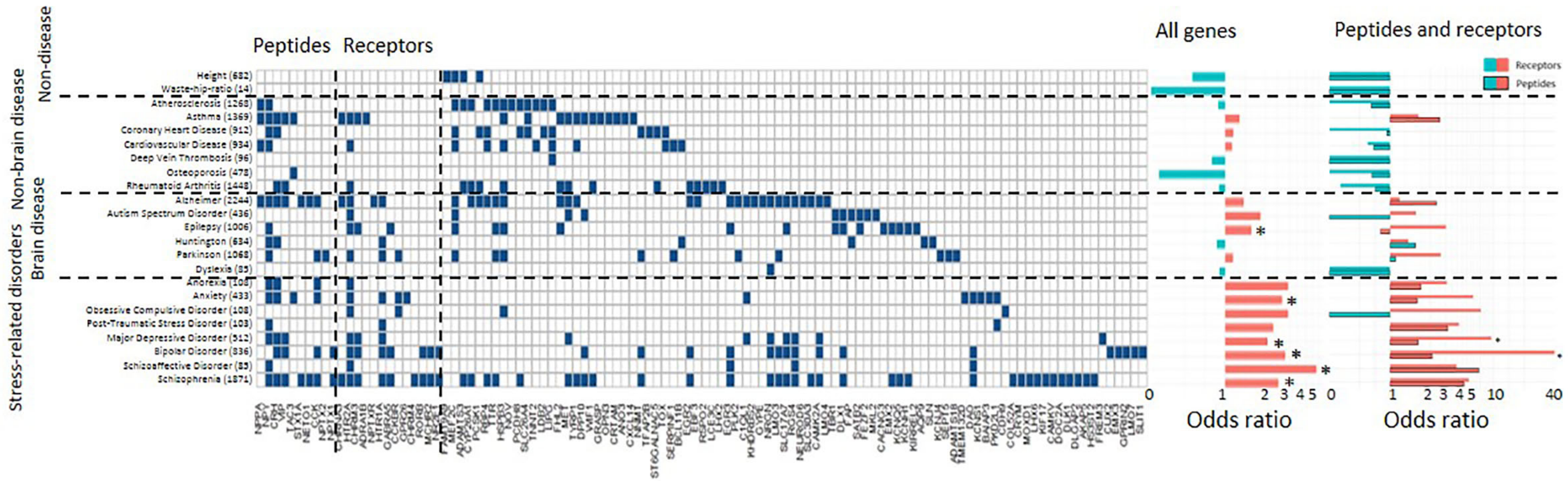
Peptides
Receptors
All genes
Peptides and receptors
Receptors
Peptides
Non-disease
Non-brain disease
Brain disease
Stress-related disorders
Height (682)
Waste-hip-ratio (14)
Atherosclerosis (1268)
Asthma (1369)
Coronary Heart Disease (912)
Cardiovascular Disease (934)
Deep Vein Thrombosis (96)
Osteoporosis (478)
Rheumatoid Arthritis (1448)
Alzheimer (2244)
Autism Spectrum Disorder (436)
Epilepsy (1006)
Huntington (634)
Parkinson (1068)
Dyslexia (85)
Anorexia (108)
Anxiety (433)
Obsessive Compulsive Disorder (108)
Post-Traumatic Stress Disorder (103)
Major Depressive Disorder (512)
Bipolar Disorder (836)
Schizoaffective Disorder (85)
Schizophrenia (1871)
0
1
2
3
4
5
Odds ratio
0
1
2
3
4
5
10
40
Odds ratio

Figure S6 Differentially expressed genes in our stress network based on siblings of schizophrenia patients and in other stress-related psychiatric disorders Disease risk gene enrichment was performed for the differentially expressed genes. The diseases are clustered as non-brain related disease, brain disease and psychiatric disease. As a none-disease-associated set of genes, waste-hip ratio and height were used. Numbers between the parenthesis indicate the number of genes known to be associated with the disease, based on DisGeNet. The effect size of the gene enrichment is presented at the middle part of the figure and considered significant when the BH-adjusted p-value < 0.05 (*). Blue bars mean depletion of genes, whereas red bars indicate enrichment of genes in a trait. ORs for the number of receptors and peptides (denoted by the black borders around the bars) in the set of differentially expressed genes are depicted for every trait (shown on the right side of the graph).

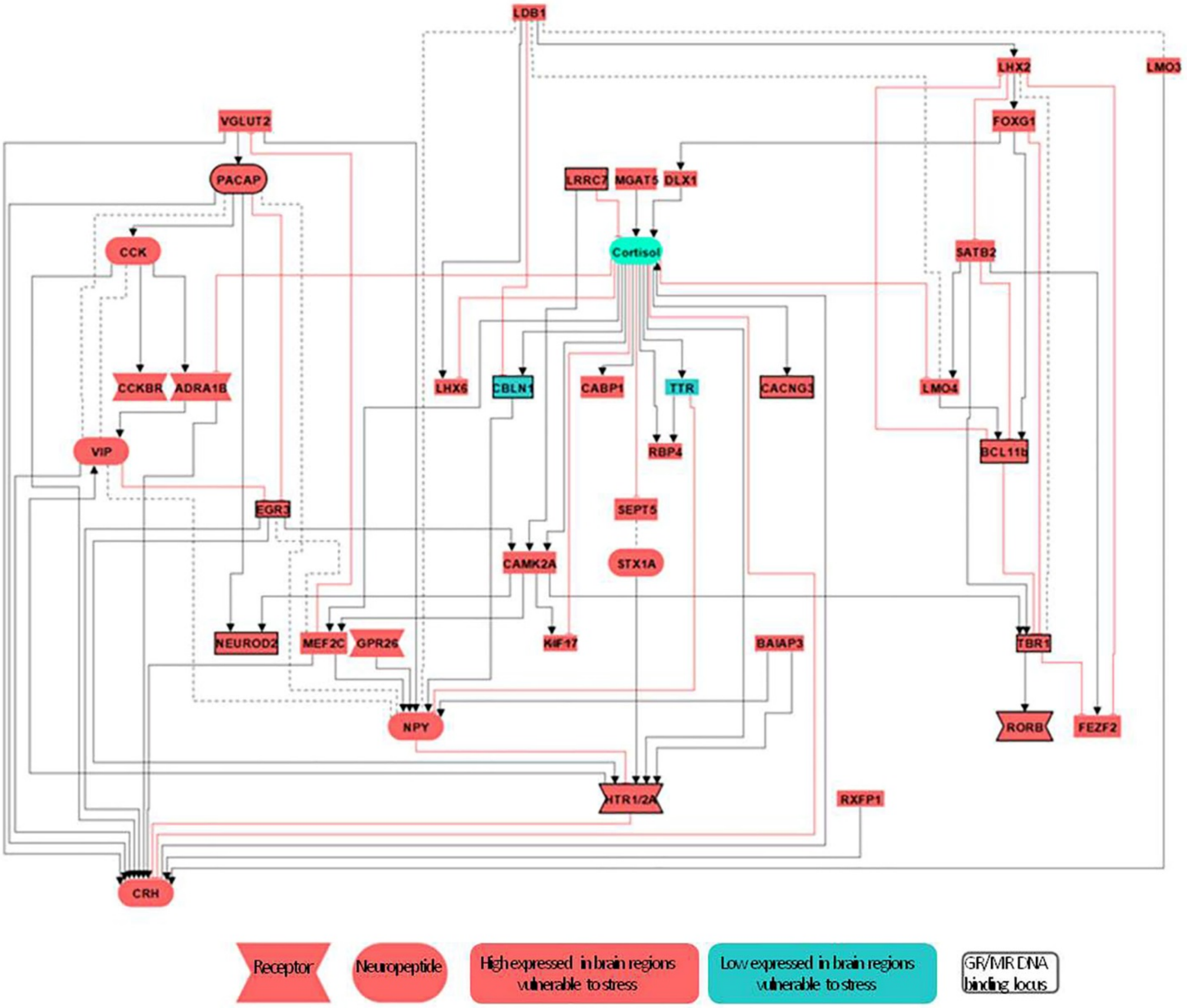

Figure S7 Putative signaling pathways as the basis of inter-individual differences in stress reactivity

Based on literature search, we assessed whether genes were known to interact with the HPA-axis and each other, resulting in a possible signaling pathway – although spatial specificity is absent from the figure. Red boxes indicate high expression levels of the differentially expressed genes in the brain regions vulnerable to stress, whereas blue boxes indicate low expression. Rounded boxes reflect neuropeptides and notched boxes reflect receptors. Squared boxes indicate the presence of a GR or MR DNA-binding loci in the vicinity of the gene. Black arrows reflect stimulation, the red t-bar indicates inhibition and the dashed line reflects binding or interaction of proteins.
